# The Diagnostic Potential of Eye Tracking to Detect Autism Spectrum Disorder in Children: A Systematic Review

**DOI:** 10.3390/medsci14010028

**Published:** 2026-01-06

**Authors:** Marcella Di Cara, Carmela De Domenico, Adriana Piccolo, Angelo Alito, Lara Costa, Angelo Quartarone, Francesca Cucinotta

**Affiliations:** 1 IRCCS Centro Neurolesi Bonino-Pulejo, S.S. 113 Via Palermo, C. da Casazza, 98124 Messina, Italy; marcella.dicara@irccsme.it (M.D.C.); carmela.dedomenico@irccsme.it (C.D.D.); adriana.piccolo@irccsme.it (A.P.); angelo.quartarone@irccsme.it (A.Q.); francesca.cucinotta@irccsme.it (F.C.); 2 Department of Biomedical, Dental Sciences and Morphological and Functional Images, University of Messina, 98122 Messina, Italy; 3 Department of Clinical and Experimental Medicine, University of Messina, 98125 Messina, Italy; lcosta@unime.it

**Keywords:** eye-tracking, autism spectrum disorder, diagnosis, visual attention, oculomotor strategies, early intervention, systematic review

## Abstract

**Background**: Autism spectrum disorder (ASD) is associated with distinct visual attention patterns that provide insight into underlying social-cognitive mechanisms. **Methods**: This systematic review (PROSPERO: CRD42023429316), conducted per PRISMA guidelines, synthesizes evidence from 14 peer-reviewed studies using eye-tracking to compare oculomotor strategies in autistic children and typically developing (TD) controls. A comprehensive literature search was conducted in PubMed, Web of Science, and Science Direct up to March 2025. Study inclusion criteria focused on ASD versus TD group comparisons in individuals under 18 years, with key metrics, fixation duration and count, spatial distribution, saccadic parameters systematically extracted. Risk of bias was assessed using the QUADAS-2 tool, revealing high heterogeneity in both index tests and patient selection. **Results**: The results indicate that autistic children exhibit reduced fixation on socially salient stimuli, atypical saccadic behavior, and more variable spatial exploration compared to controls. **Conclusions**: These oculomotor differences suggest altered mechanisms of social attention and information processing in ASD. Findings suggest that eye-tracking can contribute valuable information about heterogeneous gaze profiles in ASD, providing preliminary insight that may inform future studies to develop more sensitive diagnostic tools. This review highlights visual attention patterns as promising indicators of neurocognitive functioning in ASD.

## 1. Introduction

Autism spectrum disorder (ASD) is a complex neurodevelopmental condition for which timely and accurate diagnosis remains a significant clinical challenge, particularly regarding to early identification and personalized intervention [1,2]. According to the diagnostic criteria outlined in the DSM-5, ASD is characterized by persistent deficits in social communication and social interaction across multiple contexts, such as impairments in social reciprocity, nonverbal communicative behaviors, and the ability to develop and maintain relationships, together with restricted and repetitive patterns of behavior, interests, or activities. These features typically emerge during early developmental periods, often becoming evident before the age of three [3]. The clinical presentation can be highly heterogeneous, both in terms of severity and in its developmental trajectory over time [4,5]. This heterogeneity is framed through a three-level severity scale, Level 1 (“requiring support”), Level 2 (“requiring substantial support”), and Level 3 (“requiring very substantial support”), which captures the extent of social communication difficulties and the degree to which restricted and repetitive behaviors interfere with everyday functioning [3]. Additionally, autistic individuals may present various neurological, psychiatric, and medical comorbidities [6]; Common co-occurring conditions include intellectual developmental disorder, Attention Deficit Hyperactivity Disorder (ADHD), anxiety, epilepsy, and sleep disturbances, all of which can markedly affect daily functioning and care needs [7] Moreover, alterations in immune, inflammatory, oxidative, mitochondrial, and other metabolic processes have also been reported [8,9], adding further complexity to the clinical variability of ASD. This broad spectrum of co-occurring conditions can obscure the clinical picture and reinforces the necessity for a multidisciplinary diagnostic approach [10,11]. Currently, ASD diagnosis primarily relies on clinical judgment according to criteria established by international classification systems [3,12]. Although standardized observational instruments remain the gold standard, their use may be limited in some settings due to the time, training, and expertise required for their administration [13,14]. Evidence also shows that diagnostic certainty can vary across clinical settings [15]; nonetheless, some studies indicate that clinicians working in primary care may achieve good accuracy under specific conditions when relying on more streamlined procedures [16,17]. Complementary to these findings, telehealth-based approaches have shown promise in reducing practical barriers and waiting times, demonstrating acceptable levels of accuracy and feasibility across different contexts [18,19,20,21,22]. Timely and accurate diagnosis is a critical step to start the best intervention tailored to specific needs to improve outcomes in different developmental areas [23,24]; nevertheless, often the first signs of ASD are present before the second year of life, but formal identification and diagnosis are not confirmed until much later [25,26]. For all these reasons, understanding the neurocognitive mechanisms underlying ASD is critical for developing targeted interventions [27].

In recent years, eye-tracking metrics have emerged as a powerful, non-invasive methodology to investigate visual attention dynamics in autism and other developmental contexts [28]. This approach provides precise quantification of oculomotor behaviors such as fixation duration, saccadic movements, and gaze patterns, which reflect underlying attentional and cognitive processes [29,30,31,32].

Within this expanding body of research, numerous narrative reviews, systematic reviews, and meta-analyses have synthesized eye-tracking findings in ASD. Early reviews highlighted the relevance of eye-tracking as a tool for investigating atypical social attention and visual exploration patterns in autistic individuals, particularly during early development [33,34,35]. Subsequent systematic reviews and meta-analyses further confirmed reduced attention to social stimuli and altered gaze allocation in children with ASD, while also emphasizing substantial heterogeneity in experimental paradigms, outcome measures, and stimulus characteristics [36,37].

More recent reviews have extended this literature by focusing on specific populations or methodological approaches, including infants at high risk for ASD [38,39], autistic adults [40], and machine-learning approaches applied to eye-tracking data [41]. Additionally, recent meta-analyses have evaluated the potential role of eye-tracking as a screening or early diagnostic tool, reporting promising but still variable sensitivity and specificity across tasks and age groups [42].

Converging recent meta-analysis [29] revealed significant differences in autistic individuals compared to controls, supporting unusual gaze patterns in autistic people. In particular, reduced the studies showed that a low rate of eye fixation to social stimuli was predictive of severe ASD symptoms [32,43,44], even if it is not associated with impairment in communication, daily living skills, or socialization [45]. Furthermore, differences in social attention in ASD appear to be modulated by the presence of communicative cues such as eye contact and language [35,46,47]. Overall, previous studies have highlighted abnormalities in visual fixation patterns and gaze behaviors in people with autism, particularly reduced attention to social areas such as the face and increased attention to non-social objects. These studies have aggregated various outcomes related to eye-tracking metrics, showing results consistent with specific difficulties in regulating social attention, while maintaining a certain heterogeneity in the definition and categorization of behavioral outcomes [29,34,36].

Eye-tracking research have highlighted the importance of experimental paradigms utilizing Areas of Interest (AoI) [48]. These paradigms enable precise measurement of visual attention to specific regions within complex stimuli and offer ecologically valid assessments of attentional allocation in real-world scenarios [49]. Paradigms including static and dynamic scene viewing, the visual world, and naturalistic interaction tasks have been validated as robust tools for interpreting gaze behavior and attentional processes across diverse contexts [28]. Moreover, eye-tracking approaches are increasingly being evaluated within real-world diagnostic pathways, including primary care settings, demonstrating promising feasibility and classification performance [50,51].

Despite the substantial body of review literature, most previous syntheses have primarily aggregated results based on stimulus type, age group, or diagnostic performance, rather than organizing findings according to core behavioral domains of visual attention. As a result, the relationship between specific oculomotor behaviors and clinically meaningful aspects of ASD remains insufficiently systematized.

This systematic review examines whether oculomotor behaviors differentiating autistic children from their typically developing (TD) peers may support the identification of reliable visual attention markers. By synthesizing these behavioral differences, we aim to evaluate the diagnostic potential of eye-tracking strategies as sensitive and specific indicators of ASD. This approach may also contribute to a better understanding of the neurocognitive mechanisms underlying social behavior and inform more refined diagnostic processes.

## 2. Materials and Methods

The protocol of this systematic review was registered in the PROSPERO database (registration number: CRD42023429316). The review was conducted in accordance with the Preferred Reporting Items for Systematic Reviews and Meta-Analyses (PRISMA) guidelines [52,53], and the PRISMA-diagnostic test accuracy (DTA) statement for diagnostic test accuracy studies [54] (see Supplementary Materials Table S1), to evaluate the validity of eye tracking technology as a diagnostic measure for ASD.

### 2.1. Information Sources and Search Strategy

A comprehensive search strategy was conducted across three electronic databases: PubMed (National Library of Medicine), Science Direct (Elsevier), and Web of Science. In PubMed, the search combined MeSH terms and free-text keywords using Boolean operators (AND/OR), while ScienceDirect and Web of Science were queried using equivalent keyword combinations. The search was conducted up to 30 March 2025. Search terms were designed to capture the intersection of “autism,” “eye tracking,” and “diagnosis,” and the full strategy for each database is provided in Supplementary Materials (Supplement S1). To enhance a more comprehensive evidence base, we also performed a manual search of the reference lists of included studies. This approach is motivated by the desire to focus on studies that directly address the application of eye tracking as a diagnostic tool in ASD, ensuring targeted and specific research to evaluate the effectiveness of this technology.

### 2.2. PIRD (Population, Index Test, Reference Test, Diagnosis/Outcome)

This systematic review aimed to assess the diagnostic accuracy of eye-tracking technologies in identifying ASD, compared to standard clinical diagnostic tools. Following the PIRD framework, as outlined in the Joanna Briggs Institute methodological guidance for diagnostic test accuracy reviews [55]:

- Population: ASD Individuals;
- Index test: Eye tracking parameters;
- Reference standard: Diagnostic and Statistical Manual of Mental Disorders (DSM-IV [56] or the DSM-5 [3]) diagnostic criteria;
- Diagnostic outcome: Accuracy metrics, including sensitivity, specificity, the Area Under the Curve (AUC), and predictive values.

### 2.3. Selection Criteria

We included studies meeting the following criteria: (1) comparison between ASD and at least one TD control group; (2) participants aged under 18 years; (3) the study investigated the usability of eye-tracker parameters in characterizing and differentiating ASD-specific visual attention patterns or oculomotor behaviors from those observed in TD individuals; (4) published in a peer-reviewed journal.

Given the heterogeneity of neurodevelopmental conditions and to maintain a high degree of specificity and homogeneity in our synthesis, we focused exclusively on results pertaining to the comparison between ASD and TD groups. This approach was adopted to assess the diagnostic relevance of eye-tracking measures, namely their sensitivity and specificity in distinguishing ASD-related features from typical developmental patterns. Even when studies included additional groups beyond TD (e.g., other developmental disabilities or at-risk siblings), only the ASD vs. TD comparisons were extracted and analyzed. We included studies that employed eye-tracking methodologies to investigate visual attention and oculomotor patterns in individuals with ASD, such as fixation distribution, saccadic movements, gaze allocation to regions of interest, or temporal dynamics of visual exploration. Studies that combined pupillometry with these measures were also included, provided that the pupillary data were used alongside other eye-tracking parameters to characterize or differentiate ASD-specific profiles.

Studies were excluded if they met any of the following criteria: (1) review articles, commentaries, case reports, and editorials; (2) studies that did not compare ASD individuals with TD; (3) studies that only described clinical or neuropsychological characteristics of participants without using eye-tracking measures to compare ASD and TD peers; (4) studies focusing solely on relationships between eye-tracking data and symptom severity or other clinical scores without testing whether eye-tracking measures could discriminate ASD from TD; (5) unavailable data or full-text article; (6) studies not in the English, Spanish, German, French or Italian languages; (7) studies with overlapping samples, and when overlap was found, we included the largest study; (8) studies employing machine learning or artificial intelligence methods for classification, as these rely on internal validation (e.g., cross-validation). Finally, (9) we excluded studies that focused exclusively on pupillometry Pupillometric alterations in ASD have been extensively reviewed in recent systematic syntheses [57], and thus fell outside the scope of the present work.

### 2.4. Selection Procedures

**Figure 1:**
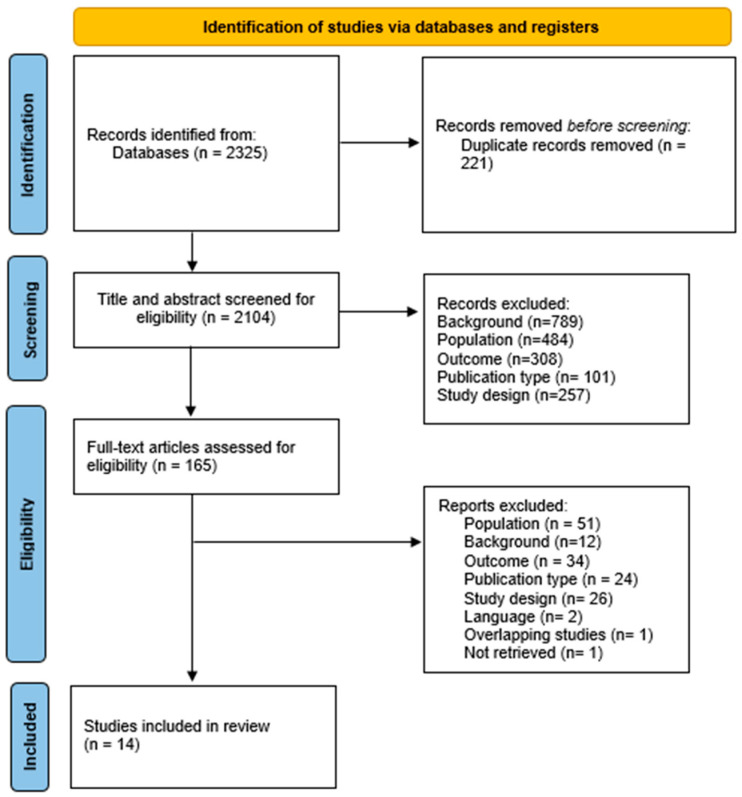
Prisma flow diagram for literature search strategy. The figure outlines the search and review process with the total number of articles included and excluded in this review.

Study selection was independently performed by two blind reviewers (M.D.C. and C.D.D.), who were blinded to each other’s based on title, abstract, and full-text content until they met to reach a final consensus. In case of disagreement, a third reviewer (F.C.) was consulted to reach consensus. If uncertainty remained, the article was moved to the next stage of evaluation. Initial screening involved excluding clearly irrelevant records based on title and abstract. During full-text review, studies were excluded if they failed to meet predefined eligibility criteria (see Section 2.2). Inter-rater agreement during the selection process was 97.7%. Reasons for exclusion at each stage are summarized in the PRISMA flow diagram (Figure 1) and Supplementary Materials (Table S1).

### 2.5. Data Extraction

Data extraction was designed to capture all variables relevant to evaluating the diagnostic accuracy and discriminative potential of oculomotor and visual attention parameters in differentiating ASD from TD peers. All variables were initially coded as categorical and well-defined by one author (F.C.), who developed the extraction framework and coding scheme. After this preliminary phase, two authors (M.D.C. and C.D.D.) independently applied the coding criteria and carried out the data extraction for all included studies. The process included both intermediate and final agreement checks to ensure consistency and reliability. Any discrepancies identified during the extraction were discussed and resolved by consensus among the authors. At the final stage, a thorough cross-checking confirmed full agreement on the coding and synthesis of the articles. Extracted information included: (a) general information comprising first author, journal and year of publication and country; (b) sample characteristics (size, sex, and age) and clinical diagnostic assessment performed; To ensure developmental consistency across studies and in line with methodological recommendations, participants’ ages were categorized into four neurodevelopmental groups during data extraction: (1) infants and toddlers (0–3 years), (2) preschool-aged children (4–5 years), (3) school-aged children (6–11 years), and (4) adolescents (12 years and older). In addition, cognitive and developmental assessment tools reported by the included studies were extracted, such as the Mullen Scales of Early Learning (MSEL), the Vineland Adaptive Behavior Scales (VABS), and the Wechsler Preschool and Primary Scale of Intelligence (WPPSI-III). (c) eye-tracking tools and the technical features of used metrics including eye tracker device, sampling rate (Hz), screen distance (cm), resolution pixel and monitor’s inches; (d) stimulus type used for eye tracking, including neuropsychological construct, task type, gaze parameters and main results. Finally, diagnostic performance data were extracted (e.g., sensitivity, specificity, accuracy, AUC, or correlation coefficients with gold-standard tools) only when explicitly reported in the included studies and derived from standardized statistical procedures such as receiver operating characteristic (ROC) curve analysis or regression models. These data were collected to support the evaluation of the potential diagnostic utility of each eye-tracking paradigm.

### 2.6. Quality Assessment

The QUADAS tool was used to assess the quality of the included studies [58]. Two independent reviewers assessed all included manuscripts (A.A and M.D.C.). Disagreements were resolved by consensus with a third reviewer (F.C.). QUADAS-2 evaluates risk of bias and applicability concerns across four key domains: (a) Patient Selection, which examines how participants were recruited and whether this could introduce selection bias; (b) Index Test, which assesses how the eye-tracking measures were applied and interpreted without knowledge of the reference standard; (c) Reference Standard, which evaluates the appropriateness and independence of the diagnostic assessment used as a benchmark (e.g., ADOS, ADI); and (d) Flow and Timing, which considers whether all participants received the same reference standard and were included in the analysis, and whether the interval between test and reference was appropriate. Each domain was rated for both risk of bias and applicability concerns as low, high, or unclear. The review-specific protocol is provided in Supplementary Materials (Supplement S2).

## 3. Results

The literature search yielded a total of n. 2325 articles. After removing n. 221 duplicates, the remaining n. 2104 records were assessed for eligibility by two independent authors (initially through abstract screening, followed by reading n. 165 full articles). Ultimately, n. 14 studies were included, see the entire selection process in Figure 1. General information comprising first author, journal, and year of publication are resumed in Table 1. The included studies in our review spanned from 2006 to 2022, covering a wide range of publication years; these were conducted in North America (n. 8, 57.14%), Europe (n. 4, 28.57%), and Asia (n. 2, 14.29%).

### 3.1. Sample Characteristic and Diagnostic Assessment

All articles compared ASD and TD populations. Sample sizes ranged from n. 24 to 1863 participants (mean 134.8), with an age spanning from 12 months of life to 17 years.

Most studies focused on younger populations. Five studies (35.71%) included exclusively infants and toddlers (0–3 years), while two studies (14.28%) focused specifically on preschool-aged children (4–5 years). Only one study included participants exclusively within the 6–11-year school-age range, and no study examined adolescents aged ≥12 years. Several studies spanned more than one developmental category. Three studies (21.43%) included samples covering both the 0–3 and 4–5 age ranges; one study involved participants across the 4–5 and 6–11 ranges; one study included both 6–11 and ≥12-year-old participants; and one study encompassed the full developmental span from infancy (0–3 years) through adolescence (≥12 years). Most ASD samples were male, with a ratio M:F ≥ 5:1 reported in 5 articles. The diagnostic criteria for inclusion of the ASD population are heterogeneous, making direct comparisons difficult, showing partial geographic differences, with greater consistency between North American and European studies and more heterogeneous diagnostic approaches in Asian samples. Most studies (*n* = 9, 56.25%) confirmed ASD diagnosis according to DSM: our studies used the DSM-IV [56], while another four studies referred to the DSM-5 [3]. One study also included International Classification of Diseases (ICD-10) criteria [59]. Moreover, among all studies, only one author did not support clinical diagnosis with standardized psychological assessment instruments. The majority of included articles used the Autism Diagnostic Observation Schedule (e.g., ADOS-2 or ADOS T or ADOS-G; n. 12, 85.71%) [60] and Autism Diagnostic Interview-Revised (ADI-R; n. 7, 50%) [61]. Several studies incorporated additional assessments to capture broader aspects of neuropsychological functioning. The Mullen Scales of Early Learning (MSEL) [62] were employed in n. 6 (42.86%) studies to evaluate cognitive development in young children, while the Vineland Adaptive Behavior Scales (VABS) [63] assessed adaptive skills necessary for daily living in n. 7 studies (50%). Other tools such as the Diagnostic Interview for Social and Communication Disorders (DISCO) [64], the Wechsler Preschool and Primary Scale of Intelligence (WPPSI-III) [65] and the Aberrant Behavior Checklist (ABC) [66] were used in one study, highlighting the different methodologies employed in assessing neurodevelopmental conditions. Although no single elective test was uniformly used across the included studies, ADOS-2 remains the most frequently employed tool for autism diagnosis by clinicians. A summary of these findings is presented in Table 2.

### 3.2. Eye-Tracking Device

Different eye-tracking devices were used in the studies reviewed. Most studies (n. 8; 57.14%) used Tobii eye tracking systems [67,68,69,70,71,72,73,74], one study used the Eyelink 1000 [75], three studies (21.43%) used the SMI iView [76,77,78], and two studies (14.29%) used another device attached to an undefined gaze tracker [79,80]. Eye-tracking sampling frequency varied across the studies: the most frequently used frequency was 60 Hz, accounting for 57.14% (n. 8) [67,69,70,74,76,78,79,80] and two studies (14.29%) used a frequency of 120 Hz [71,77]. Four studies (28.57%) used different frequencies (e.g., 50, 90, 300, 500, Hz) [68,72,73,76]. The eyes-monitor distance ranged from 50 to 90 cm, with most studies reporting ≈ 60 cm (n. 5, 35.71%) [67,68,71,75,80]. The size of the screen used ranged from 16 to 30 inches. Only five studies (35.71%) specified screen resolution [70,72,75,76,80], which varied from 1920 × 1200 (n. 4, 28.57%) [70,72,75,76] to 640 × 480 (n. 1, 7.14%) [80] pixels. These findings are summarized in Table 3.

**Table 1:** Literature References.

Author	Year	Title	Journal	Location
Anderson, C.J. et al. [79]	2006	Visual scanning and pupillary responses in young children with autism spectrum disorder	Journal of Clinical and Experimental Neuropsychology	North America—USA
Chevallier, C. et al. [67]	2015	Measuring social attention and motivation in Autism Spectrum Disorder using eye-tracking: Stimulus type matters	Autism Research	North America—Pennsylvania
Falck Ytter, T. [68]	2008	Face inversion effects in autism: a combined looking time and pupillometric study	Autism Research	Europe—Sweden
Falck Ytter, T. et al. [69]	2013	Visualization and Analysis of Eye Movement Data from Children with Typical and Atypical Development	Journal of Autism and Developmental Disorders	Europe—Sweden
Frazier, T.W. et al. [76]	2018	Development and Validation of Objective and Quantitative Eye Tracking–Based Measures of Autism Risk and Symptom Levels	Journal of the American Academy of Child & Adolescent Psychiatry	North America
Jones, W. et al. [80]	2008	Absence of Preferential Looking to the Eyes of Approaching Adults Predicts Level of Social Disability in 2-Year-Old Toddlers With Autism Spectrum Disorder	Archives of General Psychiatry	North America—Connecticut
Kou, J. et al. [70]	2019	Comparison of three different eye-tracking tasks for distinguishing autistic from typically developing children and autistic symptom severity	Autism Research	ASIA—Cina
Muratori, F. et al. [77]	2019	How Attention to Faces and Objects Changes Over Time in Toddlers with Autism Spectrum Disorders: Preliminary Evidence from An Eye Tracking Study	Brain Sciences	Europe—Italy
Pierce, K. et al. [71]	2011	Preference for Geometric Patterns Early in Life as a Risk Factor for Autism	Archives of gGeneral Ppsychiatry	North America—California
Polzer, L. et al. [72]	2022	Pupillometric measures of altered stimulus-evoked locus coeruleusnorepinephrine activity explain attenuated social attention in preschoolers with autism spectrum disorder	Autism Research	Europe—Germany
Putra, P et al. [73]	2021	Identifying autism spectrum disorder symptoms using response and gaze behavior during theGo/NoGo game CatChicken	Scientific Reports	Asia—Japan
Shic, F. et al. [75]	2022	The autism biomarkers consortium for clinical trials: evaluation of a battery of candidate eye-tracking biomarkers for use in autism clinical trials	Molecular Autism	North America—Connecticut—USA—North Carolina—Massachusetts—Washington—California
Wang, Q. et al. [78]	2018	Operationalizing atypical gaze in toddlers with autism spectrum disorders: a cohesion-based approach	Molecular Autism	North America—Connecticut
Wen, T. H et al. [74]	2022	Large scale validation of an early-age eye tracking biomarker of an autism spectrum disorder subtype	Scientific Reports	North America—California

**Table 2:** Sample characteristics and diagnostic evaluation.

Author	Sample	M:F	Years Mean (SD); [Range]	Psychodiagnostic Assessment
Anderson, C.J. et al. [79]	9 ASD ^1^	8:1	49.6 months (n.r); [12–72]	ADOS-G; MSEL
	6 DD	6:0	43.6 months (n.r); [12–72]	
	9 TD	8:1	49.8 months (n.r); [12–72]	
Chevallier, C. et al. [67]	59 ASD	13:1	12.2 years (3.3); [6–17]	DSM-IV; ADOS; ADI-R
	22 TD	22:0	14.9 years (1.7); [6–17]	
Falk Ytter, T. [68]	15 ASD	4:1	5.2 years (11 months); [n.r]	DSM-IV; ADOS; ADI-R
	15 TD	2.75:1	4.11 years (38 day); [n.r]	
Falk Ytter, T. et al. [69]	39 ASD	7:1	6.1 years (0.8); [4.0–7.3]	DSM-IV; DISCO interview; ABC; WPPSI-III; VABS
	28 TD	1:1	6.1 years (0.6); [4.3–7.3]	
Frazier, T.W. et al. [76]	91 ASD	4.6:1	5.7 years (3.6); [1.6–15.8]	DSM-5; ADOS-2; MSEL
	110 TD	3.5:1	6.8 years (3.3); [1.8–17.6]	
Jones, W. et al. [80]	15 ASD	3:1	2.28 years (0.58); [n.r]	ADOS-2; ADI-R; MSEL;VABS
	15 DD	3:1	2.06 years (0.66); [n.r]	
	36 TD	2:1	2.03 years (0.68); [n.r]	
Kou, J. et al. [70]	32 ASD	4:1	3.72 years (1.25); [n.r]	DSM-IV; ICD-10; ADOS-2
	34 TD	3:1	4.10 years (0.47); [n.r]	
Muratori, F. et al. [77]	12 ASD	5:1	T1: 25.1 months (4.6); [19–33] T2: 31.7 months (4.7); [25–39]	DSM-5; ADOS-2; VABS
	15 TD	6:1	T1: 26.5 months (4.1); [18–30]	
Pierce, K. et al. [71]	37 ASD	4:1	26.7 months (7.7); [14–42]	ADOS-T; ADI-R; MSEL; VABS
	22 DD	3:1	22.7 months (8.5); [12–41]	
	51 TD	2:1	24.6 months (8.2); [12–43]	
Polzer, L. et al. [72]	57 ASD	1.6:1	47.95 months (10.21); [18–65]	DSM-5; ADOS2; ADI-R
	39 TD	1:1	33.28 months (10.31); [18–65]	
Putra, P. et al. [73]	21 ASD (10 with ADHD)	2:1	4.6 years (0.4); [n.r.]	diagnosed by clinicians
	31 TD	3:1	5 years (0.6); [n.r.]	
Shic, F. et al. [75]	280 ASD	3:1	T1: 8.55 years (1.64); [6–11.6] T2: + 6 week	DSM-5; ADOS-2; ADI-R; VABS
	119 TD	2:1	T1: 8.51 years (1.61); [6–11.6] T2: + 6 week	
Wang, Q. et al. [78]	112 ASD	6:1	22.39 months (3.02); [n.r]	ADOS-G; ADI-R; MSEL; VABS
	36 DD	8:1	21.71 months (3.38); [n.r]	
	163 TD	1:1	21.89 months (3.39); [n.r]	
Wen, T.H. et al. [74]	725 ASD	3:1	26.40 months (8.25); [12–49]	ADOS-2; MSEL; VABS
	103 ASD-Feat	5:1	23.84 months (9.17); [11–44]	
	128 GDD	3:1	26.38 months (9.81); [12–46]	
	198 LD	3:1	20.78 months (7.44); [10–48]	
	162 Other	2:1	23.15 months (9.31); [11–48]	
	487 TD	2:1	23.32 months (9.17); [11–48]	
	60 TypSIB ASD	1:1	21.86 months (8.79); [12–44]	

^1^ ASD: Autism Spectrum Disorder; TD: Typically Developing; ADHD: Attention Deficit Hyperactivity Disorder; ASD-feat: ASD features; GDD: Global developmental delay; LD: language delay; TypSibASD: typical toddlers with an ASD sibling; DD: developmentally delayed; ADOS: Autism Diagnostic Observation Schedule; ADI: Autism Diagnostic Interview; DSM: diagnostic and statistical manual of mental disorders; MSEL: Mullen Scales of Early Learning; VABS: Vineland Adaptive Behavior Scales; ABC: Autistic Behavior Checklist; WPPSI: Wechsler Preschool and Primary Scale of Intelligence; ICD: International Classification of Diseases; n.r: not reported.

**Table 3:** Eye tracker device specifications.

Author	Eye Tracker Device	Sampling Rate (Hz)	Screen Distance (cm)	Resolution Pixel	Monitor’s Inches
Anderson, C.J. et al. [79]	ASL Model 504 (Applied Science Laboratories)+ Flock of Birds magnetic head tracker	60 Hz	n.r	n.r	16″
Chevallier, C. et al. [67]	Tobii X120	60 Hz	60	n.r	30″
Falck Ytter, T. [68]	Tobii 1750 remote infrared eye tracker	50 Hz	60	n.r	17″
Falck Ytter, T. et al. [69]	Tobii T120	60 Hz	n.r	n.r	n.r
Frazier, T.W. et al. [76]	SMI RED 250	60 Hz	75	1280 × 1024	19″
Jones, W. et al. [80]	ISCAN Inc	60 Hz	50.8	640 × 480	20″
Kou, J. et al. [70]	Tobii TX300 Binocular	60 Hz	n.r	1920 × 1080	23″
Muratori, F. et al. [77]	SMI RED 500	120 Hz	50	n.r	n.r
Pierce, K. et al. [71]	Tobii T120	120 Hz	60	n.r	17″
Polzer, L. et al. [72]	Tobii TX300	300 Hz	50–80	1920 × 1080	n.r
Putra, P et al. [73]	Tobii 4C	90 Hz	50–90	n.r	n.r
Shic, F. et al. [75]	Eyelink 1000 Plus binocular remote	500 Hz	65	1920 × 1200	24″
Wang, Q. et al. [78]	SMI iView X RED	60 Hz	75	n.r	24″
Wen, T. H et al. [74]	Tobii T120	60 Hz	n.r	n.r	n.r

n.r: not reported.

### 3.3. Psychophysics and Neuropsychological Constructs

Nine studies (64.29%) utilized a preferential-looking paradigm [67,68,70,71,72,74,75,79,80] an experimental method designed to compare gaze fixation across different stimuli. Among those, two studies used both the activity monitoring and preferential-looking paradigm [67,75]. Overall, preferential-looking paradigm was sensitive to differences between populations mainly for samples younger than 9 years of age. Moreover, three studies (21.43%) focused on the activity monitoring paradigm [67,75,78], which explores how visual attention is allocated to stimuli in intricate social scenes involving shared activities. One of these studies used also include the task of joint attention [78], to assess initiation and response to joint attention, without significant results. However, studies focusing on joint attention paradigms consistently reported atypical gaze behavior in autistic children, including reduced gaze alternations, lower synchronization with social stimuli, and altered attention to joint targets. This paradigm is reported in one study [77]. These findings highlight early impairments in join attention mechanisms, a core domain affected in ASD. Two studies (14.29%) analyzed the social information processing paradigm [69,76], which refers to how people process social information to understand and respond to social situations. One study (7.14%) used different paradigms: inhibiting action and spatial and gaze adjustment [73]. See Table 4 for details.

### 3.4. Type of Stimuli

The tasks used in each study were evaluated and ranked to assess their usefulness for diagnosis. A wide heterogeneity was observed in the types of stimuli adopted across the reviewed studies, including both dynamic and static visual stimuli. The number and order of stimulus presentations varied among the studies, with most presenting stimuli randomly.

Of the reviewed studies, n. 7 (50%) used dynamic stimuli [68,69,71,74,77,78,80], n. 2 (14.29%) static stimuli [73,79], and n. 5 (35.71%) employed both dynamic and static stimuli [67,70,72,75,76]. One study focused on social tasks [68], and one study used a non-social task [73], the remaining studies (n. 12; 85.71%) utilized both types of tasks [67,69,70,71,72,74,75,76,77,78,79,80]. Most studies (n. 11, 78.57%) presented task in a passive format in which participants observe the visual stimuli without producing any behavioral response [68,69,70,71,72,74,75,76,77,78,79]. Only two studies (14.29%) included an active mode of presentation, that require an explicit, goal-directed response from the participant during stimulus presentation [73,80], and one study employed both modalities [67]. The results of these characteristics are summarized in Table 4.

### 3.5. Outcome Parameters and Metrics of the Eye Tracker

#### 3.5.1. Fixation Based Measures

Fixation, defined as the maintenance of gaze on a specific AOI for a measurable period, represents one of the most extensively examined oculomotor parameters in eye-tracking research. All the included studies (n. 14; 100%) employed at least one fixation-related measure for descriptive, classificatory, or diagnostic purposes. Among these, temporal fixation metrics, such as total fixation duration, mean fixation time, or fixation rate, were the most frequently reported, appearing in 11 studies (78.6%) [67,68,69,70,71,72,73,76,77,79,80] followed closely by proportion-based fixation measures in 11 studies (78.6%) [67,68,69,70,71,72,74,75,76,77,80], which quantify the relative allocation of gaze across competing AOIs. Derived or composite fixation indices, integrating multiple fixation features into higher-order constructs such as ratios, difference scores, or entropy measures, were likewise identified in 11 studies (78.6%) [67,68,69,70,71,72,73,74,75,77,78]. Fixation count and transition metrics, reflecting the number and sequencing of discrete fixations, were reported in 3 studies (21.4%) [68,76,77], while spatial gaze parameters, describing the dispersion and organization of gaze within the visual field, appeared in 3 studies (21.4%) [68,73,78].

##### Temporal Fixation Metrics

Temporal fixation measures were the most commonly employed parameters, reported in 11 of the 14 studies (78.6%) [67,68,69,70,71,72,73,76,77,79,80]. These metrics quantify the duration of gaze maintenance on specific AOIs, including total fixation time, average fixation duration, and fixation rate.

Across these studies, autistic people consistently showed reduced fixation duration on socially relevant stimuli compared to TD peers, emerging as the most consistent finding. Anderson et al. [79] and Chevallier et al. [67] reported shorter total fixation times to faces and social scenes in ASD, while Falck-Ytter et al. [69] and Kou et al. [70] confirmed less stable and shorter fixations in autistic children during dynamic or interactive contexts. Frazier et al. [76] extended this observation by differentiating between first and mean fixation durations, identifying systematic group differences in ASD across AOIs. Putra et al. [73] and Muratori et al. [77] further associated reduced fixation persistence with atypical joint attention and gaze–object coordination.

Other studies [71,72,80] reinforced these trends within diverse paradigms, demonstrating consistently shorter or less sustained gaze among autistic participants, regardless of stimulus type or task complexity. Even when overall viewing duration did not differ significantly, as in Falck-Ytter [68], the temporal organization of fixations in ASD revealed altered attentional engagement. Collectively, temporal fixation measures represent the most stable and replicable indicators of gaze persistence and attentional modulation anomalies in ASD.

##### Proportion-Based Fixation

Proportion-based fixation measures were reported in 10 of the 14 studies (71.43%) [67,68,69,70,71,72,74,76,77,80]. These indices quantify the relative allocation of fixation time across competing AOIs, expressing gaze behavior as a percentage or proportion of total fixation duration directed toward a given stimulus category. Across studies, autistic participants consistently showed reduced proportional attention to socially relevant stimuli, reflecting attenuated social attentional bias

Chevallier et al. [67] introduced the Proportion of Total Fixation Duration, dividing fixation time devoted to each AOI group (social vs. object) by total on-screen fixation time, revealing reduced social preference and lower social prioritization scores in ASD. Falck-Ytter [68] computed ratios of looking time between facial regions (eyes and mouth), showing that autistic participants allocated a smaller proportion of viewing time to the eyes compared to TD peers.

In Falck-Ytter et al. [69], proportion measures were used to quantify relative fixation duration on faces, toys, and background, demonstrated reduced proportional attention to faces in ASD despite comparable total looking times.

Frazier et al. (2018) [76] employed fixation duration percent, reflecting the share of fixation time within each region of interest (RoI) relative to the total stimulus period, identifying lower fixation percentages to socially relevant regions. Similarly, Jones et al. [80] analyzed proportional fixation on eyes, mouth, and objects, showing that reduced fixation time to eyes, rather than overall looking, best discriminated diagnostic groups. Kou et al. [70] measured the mean proportion of fixation time devoted to social versus non-social stimuli across multiple dynamic tasks, reporting a consistent reduction in social preference among autistic children. Muratori et al. [77] calculated the proportion of fixation duration on each AOI (face, target object, non-target object), normalized by total fixation time, to assess joint attention performance, finding lower proportional gaze toward faces in ASD. Pierce et al. [71] and Wen et al. [74] used the percentage of fixation time within dynamic geometric images (DGI) as a diagnostic indicator, with autistic toddlers showing a high proportion of gaze toward geometric rather than social scenes. Polzer et al. [72] computed the percentage of total gaze time spent on social motion (SOC-M) versus geometric motion (GEO-M), deriving a preference score that highlighted decreased social attention in ASD.

##### Fixation and Transition Count Measures

Fixation and transition count measures were reported in 3 studies (21.4%) [69,76,77]. These indices quantify the number of discrete fixation events or transitions between AOIs, reflecting gaze fragmentation, attentional shifting, and exploratory scanning behavior.

Frazier et al. [76] and Falck-Ytter et al. [69] showed that although fixation counts were comparable, children with ASD demonstrated altered temporal distribution of fixations, suggesting different attentional switching patterns. Muratori et al. [77] analyzed transitions between face and object AOIs, reporting a reduced alternation frequency in ASD during joint attention episodes, suggesting less coordinated social attention.

##### Spatial Gaze Parameters

Spatial gaze parameters were reported in 3 studies (21.4%) [69,73,78]. These measures describe the spatial organization and dispersion of fixation patterns across visual scenes, including fixation distance from reference points, spatial variability, and gaze entropy.

Falck-Ytter et al. [69] introduced the Distance-to-Reference (D2R) metric to quantify the Euclidean distance between each fixation and a predefined social anchor point (the center of the girl’s face). Using this measure, the authors demonstrated that children with ASD consistently fixated farther away from central facial regions, showing more peripheral and spatially dispersed gaze patterns compared to typically developing peers. TD children rapidly shifted their fixations toward the girl’s face immediately after the onset of a communicative gesture, whereas ASD children showed a delayed, weaker, and less consistent orienting response, maintaining greater D2R values throughout the critical 4–6 s interval. Putra et al. [73] reported that autistic children showed markedly less structured and more variable gaze trajectories, characterized by increased spatial irregularity and reduced predictability in gaze–object tracking compared to typically developing peers. Specifically, ASD participants exhibited higher gaze-to-object entropy, greater variability in gaze acceleration, and larger fluctuations in fixation distance, reflecting unstable and disorganized spatial scanning patterns.

Similarly, Wang et al. [78] demonstrated that ASD toddlers show reduced inter-participant spatial cohesion, meaning that their gaze points were less synchronized with those of typically developing peers during moments of high attentional convergence. Using a cohesion-based normative model, the authors quantified typicality as the proximity of an individual’s gaze to the TD group during high-cohesion time frames. ASD toddlers exhibited significantly lower gaze typicality scores in socially rich contexts such as Dyadic Bid and Sandwich-Making scenes, reflecting greater spatial dispersion and weaker alignment to social focal points.

##### Derived or Composite Fixation Indices

Derived or composite fixation indices were reported in 11 studies (78.6%) [67,68,69,70,71,72,73,74,75,77,78]. These metrics combine primary fixation variables into higher-order constructs, such as difference scores, proportional ratios, entropy measures, or multivariate composite indices, that capture more abstract dimensions of gaze organization. Chevallier et al. [67] showed that autistic children exhibited significantly lower social preference scores, with reduced fixation ratios for social vs. object stimuli, indicating attenuated prioritization of social information. Falck-Ytter [68] demonstrated that ASD toddlers showed reduced preferential looking toward upright biological motion relative to TD peers.

Falck-Ytter et al. [69] further reported that autistic children fixated farther from socially salient facial regions, particularly during key communicative moments, reflecting weaker social orienting responses. Shic et al. [75] introduced the Oculomotor Index of Gaze to Human Faces (OMI), showing that autistic toddlers displayed significantly lower composite face-directed gaze scores across tasks, reflecting pervasive reductions in social attention. Wang et al. [78] developed the Typicality Score, quantifying the similarity of each child’s gaze to a normative TD cohesion model. ASD toddlers showed substantially lower typicality, particularly during Dyadic Bid and Sandwich-making scenes, and these scores correlated with greater autism symptom severity, indicating clinically meaningful gaze atypicality. Putra et al. [73] showed that autistic children exhibited higher entropy of gaze-to-object distance and angle, greater variability in gaze acceleration, and more irregular tracking of the moving target, indicating less predictable and less structured visual exploration.

Complementary derived approaches were also used by Kou et al. [70], who calculated proportional fixation indices and mean fixation durations and reported markedly reduced social preference and increased attention to geometric patterns in autistic children, with all three paradigms effectively differentiating ASD from TD group. s. Wen et al. [74] further expanded composite fixation approaches by integrating percent fixation to geometric images (DGI), optimal diagnostic thresholds (61–69%), and additional gaze-derived metrics within a multivariate classification framework. Their composite model demonstrated that percent DGI fixation alone achieved very high specificity (95–98%) and strong predictive value, and that combining fixation proportion with secondary gaze indices substantially enhanced classification performance. Muratori et al. [77] computed normalized transition scores reflecting the balance of gaze shifts between target and non-target AOIs in joint-attention tasks and found atypical transition patterns in toddlers with ASD, who showed altered disengagement and exploration compared to TD peers. Pierce et al. [71], used fixation difference ratios between geometric and social scenes as diagnostic thresholds, showing that toddlers with ASD spent significantly more time on geometric motion, with geometric fixation >69% yielding a 100% positive predictive value for ASD classification. Polzer et al. [72], derived gaze preference scores quantifying time allocated to social versus non-social motion and observed a reduced social motion preference in ASD relative to TD children, consistent with attenuated social attention patterns.

#### 3.5.2. Saccade Latency

Three studies have examined saccades in ASD. Wen et al. [74] analyzed saccadic frequency during the observation of both geometric and social stimuli. The researchers found that children with ASD who preferred geometric images had significantly fewer saccades while viewing these images than all other groups. In contrast, these children showed significantly more saccades while viewing social stimuli. A similar result was found by Pierce et al. [71] in which it was shown that autistic children who preferred geometric pictures had significantly fewer saccades per second while viewing these pictures and a higher rate of saccades while viewing non-preferred social pictures. On the other hand, autistic people who preferred social pictures showed almost typical saccade patterns. Jones et al. [80] found that overall saccadic behavior did not differ between ASD and TD toddlers; however, the ASD subgroup with a strong geometric preference showed a distinctive pattern, producing significantly fewer saccades when viewing geometric images and significantly more saccades when viewing social images.

### 3.6. Accuracy, Sensitivity, and Specificity Task

A range of eye-tracking studies has explored the diagnostic validity of various visual attention metrics in distinguishing autistic children from TD controls and other clinical populations. Across paradigms, several studies have reported high diagnostic accuracy, while others demonstrated moderate performance or focused on their utility for stratification rather than direct classification.

Early investigations such as Anderson et al. [79] demonstrated that fixation-derived parameters—including total fixation time and mean fixation duration—could differentiate autistic from typically developing participants with overall classification accuracy around 78%, establishing the feasibility of gaze-based discrimination using relatively simple visual scanning metrics.

High diagnostic performance was reported in studies employing composite gaze indices hat integrated multiple eye-tracking features or sophisticated task designs. Frazier et al. [76] achieved AUCs of 0.92 (training) and 0.86 (test), with robust performance in both younger and older children. The GeoPref Test, used by Pierce et al. [71] and Wen et al. [74], demonstrated excellent specificity (98%) but lower sensitivity (17–33%), which improved slightly when combining saccade frequency with fixation preference.

Other studies provided strong AUC values using single or dual-metric models. Putra et al. [73] achieved an accuracy of 88.6% with an AUC > 0.85, and Falck-Ytter et al. [69] reported AUCs of 0.916, 0.855, and 0.815 depending on the spatial metric employed. Earlier work by Falck-Ytter [68], although not explicitly diagnostic in design, found that ratios of fixation duration toward eyes versus mouth robustly differentiated ASD and TD groups, highlighting early evidence that proportional fixation patterns carry diagnostic relevance. Studies like Polzer et al. [72] and Wang Q. et al. [78] employed regression-based models, which demonstrated good predictive performance (e.g., pseudo R^2^ = 0.486) based on stimulus preference or gaze typicality. Kou et al. [70] also supported discriminative utility through ROC analyses with AUC values ranging from 0.714 to 0.815 depending on the task and parameter (e.g., fixation count or duration). These findings were accompanied by significant correlations with ADOS-2 severity scores, reinforcing the clinical relevance of the metrics. Muratori et al. [77] used eye-tracking measures longitudinally, showing that specific visual attention patterns (e.g., transitions from face to non-target object) predicted changes in ADOS social-communicative behaviors over time (adj-R^2^ = 0.34), suggesting moderate predictive power for developmental trajectories.

Other studies demonstrated moderate but informative performance. Chevallier et al. [67] reported a significant AUC of 0.721 only for the Interactive task, while static and dynamic tasks failed to show discriminative validity. Jones et al. [80] found that fixation on the eyes was the strongest predictor of group membership, with Cohen’s d = 1.56 (ASD vs. TD) and a significant correlation with social disability (r = –0.669), supporting its value as a unidimensional marker.

Finally, studies like Shic et al. [75] while not intended for clinical diagnosis per se, showed moderate AUCs (e.g., 0.730) and provided value for phenotypic stratification, cultural adaptation, and early screening applications.

Taken together, these results highlight the potential of eye-tracking measures to serve as objective, scalable, and sensitive tools for early identification and stratification in ASD. While sensitivity is variable, specificity and predictive power are often high, particularly when combining multiple gaze metrics or when models are trained on well-characterized clinical populations. In summary, across the included studies, the strongest diagnostic evidence emerged for proportional fixation indices, particularly social versus non-social fixation ratios, which consistently distinguished ASD from TD groups with high specificity. Temporal fixation metrics, such as total fixation duration and mean fixation time on socially salient regions, also showed robust discriminatory power and were among the most sensitive indicators of reduced social engagement. Composite and entropy-based indices, including gaze typicality scores and measures of fixation sequence regularity, further enhanced classification accuracy by capturing higher-order features of gaze organization. Additionally, saccadic patterns proved useful for differentiating subgroups within the ASD population: children with a geometric preference demonstrated distinct saccade frequency profiles compared to those showing social preference, supporting their value for stratification. Together, these metrics represent the measures with the clearest and most replicable evidence for diagnostic utility and for distinguishing phenotypic subgroups within ASD.

### 3.7. Quality of the Studies

**Figure 2:**
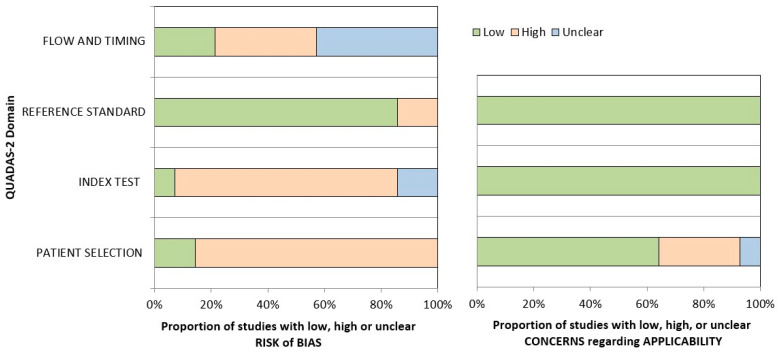
Risk of bias and applicability concerns by QUADAS-2 domain. The proportion of studies with a low, high or unclear risk of bias (**left**) and applicability concerns (**right**) is shown across the four QUADAS-2 domains.

A review of bias within the included studies was conducted using the Quality Assessment of Diagnostic Accuracy Studies (QUADAS-2) tool. Twelve out of the fourteen studies included were at high risk of bias due to patient selection (e.g., non-consecutive or unclear sampling), and eleven were at high risk in the index test (primarily due to lack of blinding or non-standardized procedures). Only two studies showed high risk for the reference standard. In the ‘Flow and Timing’ domain, five trials showed a high risk of bias, six had unclear risk, and only three showed a low risk. Most of the included trials had low concerns regarding applicability. However, four trials had a high risk in the patient selection domain, and one had an unclear risk. Although most studies had low applicability concerns, the high risk of bias in key domains undermines confidence in the overall diagnostic estimates and calls for caution when interpreting results. A comprehensive graphical representation of the analysis is shown in Figure 2 and the Supplementary Materials (Table S2).

## 4. Discussion

In recent years, eye-tracking has gained growing attention as a potential diagnostic tool due to its non-invasive nature and objective measurement capabilities [81]. To our knowledge, this is the first systematic review that comprehensively examines the use of eye-tracking to characterize oculomotor strategies distinguishing autistic children from their typically developing peers. By simultaneously evaluating the quality of experiments, the diversity of neuropsychological paradigms, types of stimuli, and gaze-related measures, this review aims to provide an integrated understanding of how visual attention patterns differ in ASD.

**Figure 3:**
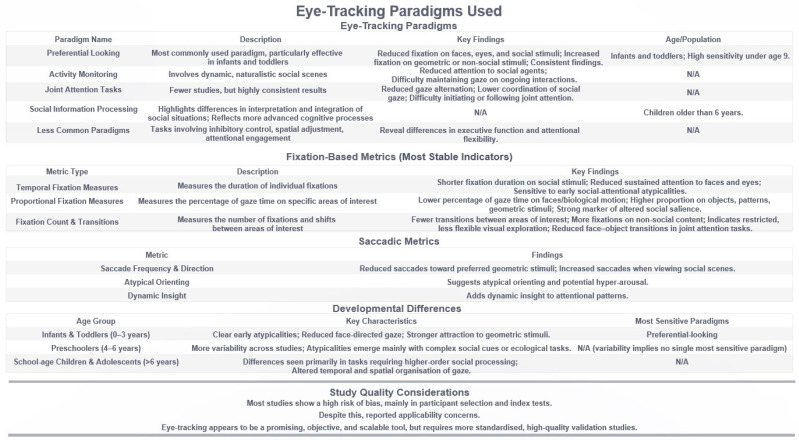
This overview summarizes the key paradigms and gaze metrics reported in the included studies, as well as the age-related differences. The findings relate to atypicalities in social attention in children with ASD, as well as considerations regarding the quality of the studies.

Figure 3 shows a summary of the experimental paradigms and eye-tracking measures used in the included studies. These paradigms span different age groups and target various domains of social and non-social visual attention in ASD.

This study also provides a preliminary synthesis of the potential diagnostic performance of eye-tracking paradigms in identifying ASD. While the pooled sensitivity (35.9%) was lower than expected, the wide confidence interval reflects the substantial uncertainty, largely due by the small number of studies and the inclusion of one study with artificially optimized specificity [74]. In contrast, the pooled specificity of 92.3% confirms the high discriminative value of gaze-based metrics in ruling out ASD in non-autistic children.

Overall, the findings suggest that gaze-based measures hold potential as a diagnostic adjunct, but also emphasize the need for larger, prospective studies to enhance generalizability and establish consistent methodological standards. Several studies have reported atypical gaze patterns in ASD children, suggesting that deviations in visual attention may be closely related to differences in social information processing [82]. These considerations align with the broader evidence underscoring the importance of early intervention in ASD, which can significantly improve developmental outcomes, enhance adaptive behaviors, and reduce symptom severity over time [83]. Implementing therapeutic strategies at an early stage can take advantage of neurodevelopmental plasticity, leading to a better long-term prognosis and quality of life [84]. However, although the literature on eye-tracking in ASD is increasing, there seems to be an apparent convergence of results, although measured differently by the various studies examined. Differences across studies likely reflect not only the intrinsic heterogeneity of ASD, but also substantial variability in experimental paradigms and technological approaches. Such variability includes differences in task structure (e.g., preferential-looking, social interaction, joint attention tasks), types of stimuli, calibration procedures, sampling rates, and eye-tracking hardware, all of which meaningfully influence gaze metrics and limit direct comparability across studies. Additional methodological challenges further undermine interpretability. Many studies employ small samples or without neurotypical controls [29,76,85], and the considerable heterogeneity in the task choice [82,86] and gaze parameters contributes to inconsistent finding. s This methodological variability complicates cross-study comparison and limits the ability to derive a unified theoretical framework for interpreting gaze differences in ASD. Across the included studies, ASD was largely treated as a homogeneous diagnostic category, with limited attention to individual variability in gaze patterns. Only a few studies (such as those examining GeoPref subgroups) attempted to identify distinct gaze-based phenotypes, and these approaches were not extended across other paradigms. Moreover, none of the studies conducted formal analyses of potential mediators such as IQ, language ability, sensory responsivity, or co-occurring conditions (e.g., ADHD, anxiety). These factors were rarely reported or incorporated into statistical models, preventing the examination of how individual neuropsychological profiles might modulate gaze behavior. Consequently, the current evidence does not allow the development of a refined theoretical model that captures the heterogeneity of autistic visual attention profiles, thereby limiting the depth of interpretation offered by existing research.

In addition, the studies included in this review conceptualized atypical gaze as an indicator of reduced social attention or altered social information processing. None of the studies interpreted gaze patterns as adaptive responses to hyper-arousal or sensory overload, perspectives increasingly acknowledged in contemporary neurodiversity-oriented frameworks [87].

Moreover, all the experiments included are heterogeneous by number, age, and sex of participants, as well as by neuropsychological paradigm and type of measurement. Although it is difficult to establish a linear organization, the results suggest that some eye-tracking measures could be used to support the diagnosis of autism. In addition, when examining age distribution within each paradigm, most preferential-looking studies focused on infants and toddlers (0–3 years), and consistently reported early-emerging atypicalities in social attention. Activity-monitoring and joint-attention paradigms were applied mainly in preschool-age children (4–6 years), with findings showing more variable effects. By contrast, social-information-processing paradigms were used almost exclusively in school-age samples, capturing more complex alterations in gaze organization. Thus, although formal age-by-group interactions could not be tested, the pattern of available studies suggests that different paradigms map onto different developmental stages, which limits direct comparability but provides preliminary insight into developmental trajectories. Furthermore, from the complexity of all the studies included emerged the need to address several essential method issues, to elaborate more consistent and comparable data for future studies. In addition to potential diagnostic performance, the practical feasibility of eye-tracking procedures should also be considered. Although eye-tracking systems involve an initial equipment cost and require trained personnel for setup and calibration, the time needed for data acquisition is relatively short, particularly in young children for whom preferential-looking paradigms can be completed within a few minutes [88,89]. Older children and adolescents can tolerate longer and more complex tasks, allowing for the collection of richer datasets without substantially increasing testing burden [90]. Importantly, eye-tracking assessments are generally well accepted by autistic children due to their non-invasive, passive, and visually engaging nature. Compliance is usually high, although sensory sensitivities or attentional fluctuations may affect performance in a minority of cases. Overall, the combination of low per-session time, high acceptability, and objective measurement supports the feasibility of integrating eye-tracking paradigms into clinical workflows [91,92,93].

### 4.1. Neuropsychological Construct and Type of Stimuli

The review of the experimental paradigms employed across studies reveals important insights into methodological diversity and their differential sensitivity in capturing atypical gaze behaviors in autistic children. The preferential-looking paradigm emerged as the most widely adopted approach, used in nearly half of the studies (43.75%). Its simplicity and suitability for young children make it particularly effective in early developmental stages [89]. This paradigm was especially sensitive in detecting group differences in children under the age of 9, suggesting its strength in early screening contexts. Its combination with other paradigms, such as activity monitoring tasks, further enhanced its capacity to capture nuanced attentional and physiological responses.

Despite its strengths, the preferential-looking approach typically captures visual preference rather than spontaneous social attention in dynamic contexts. To address this, several studies employed the activity monitoring paradigm, which involves more ecologically valid, complex social scenes. Although used less frequently (18.75%), this paradigm provided richer insights into how attention is distributed in naturalistic settings, such as shared play or collaborative tasks. While one study included a joint attention task without statistically significant findings, studies focused more explicitly on joint attention paradigms consistently demonstrated atypical gaze patterns in autistic children. These included reduced gaze alternation, lower synchronization with socially relevant stimuli, and difficulty maintaining attention on shared targets, all of which point to core impairments in the development of shared intentionality and the foundations of social communication [94,95,96,97].

Across the included studies, age-related differences emerged in how visual attention to social scenes was expressed. In infants and toddlers, preferential-looking and activity-monitoring paradigms consistently detected early atypicalities, including reduced attention to faces and a stronger focus on non-social or geometric stimuli. In preschool and early school-age children, findings were more variable and tended to appear mainly in paradigms with explicit or highly ecological social cues. In older children and adolescents, atypicalities were observed primarily in tasks requiring higher-order social information processing, reflected in altered temporal and spatial gaze patterns. Overall, these developmental variations indicate that visual attention in complex social contexts changes substantially across age, limiting the comparability of studies spanning broad developmental ranges [98]. A smaller subset of studies (25%) investigated gaze behavior through the social information processing paradigm, which assesses how individuals perceive and interpret social situations. While these paradigms are conceptually valuable, they are more cognitively demanding and thus more suitable for older children or individuals with higher developmental functioning [82]. Nonetheless, the findings from this group contributed to a broader understanding of how gaze behavior in ASD is shaped not only by perceptual salience but also by cognitive interpretation of social scenarios.

Finally, two studies (12.5%) employed less common but theoretically relevant paradigms, such as attentional engagement tasks or inhibitory control/spatial adjustment paradigms. Though less frequently used, these approaches introduced novel angles for interpreting gaze behavior, particularly regarding executive functions and attentional flexibility, which are often impacted in ASD [91].

It is also important to note that several studies relied on low-resolution eye-tracking systems or heterogeneous recording setups, which may reduce fixation accuracy, especially in young children, and could have contributed to variability. In addition, unmeasured individual factors may also influence gaze metrics and could partly account for some of the observed group differences [99]. Moreover, the included studies were conducted almost exclusively in Western or East Asian, middle-class populations, limiting generalizability, as cultural norms regarding eye contact vary substantially and may influence gaze behavior independently of ASD [100]. In summary, while preferential-looking paradigms remain a cornerstone of early gaze research due to their accessibility and sensitivity in young children, combining multiple paradigms or employing more dynamic and socially complex tasks appears to provide a richer, multi-dimensional profile of visual attention in ASD. These findings underscore the importance of task selection in eye-tracking research and highlight the potential of task-based stratification in both research and clinical applications.

### 4.2. Outcome Parameters and Gaze Metrics

The analysis of outcome parameters across included studies underscores the centrality of fixation-based metrics in delineating visual attention patterns in ASD. Among these, temporal fixation measures, including total fixation duration, average fixation time, and fixation rate, emerged as the most consistently adopted indicators across studies. Their sensitivity in capturing differences in social attention reflects the temporal dynamics of gaze engagement [101,102]. The recurrent observation of shorter fixation durations on socially salient stimuli among autistic individuals aligns with core clinical characteristics of ASD, such as diminished social interest and reduced responsiveness to dynamic interpersonal cues [103]. Evidence from naturalistic and joint attention paradigms further indicates that task complexity modulates fixation persistence, emphasizing the need to consider contextual and stimulus-related factors when interpreting temporal fixation outcomes [104]. Proportion-based fixation metrics, widely employed in visual preference paradigms, provide a normalized index of attentional allocation across competing stimuli [105]. These measures expressed as the percentage of total fixation time directed to specific AOIs proved particularly effective in differentiating social from non-social preferences [106]. Across multiple studies, autistic participants demonstrated a reduced proportion of fixation time on faces or biological motion and a corresponding increase on geometric or object-based stimuli, reinforcing the notion of an altered social salience hierarchy [71,107]. As such, proportional fixation indices represent a robust and interpretable measure of attentional bias, suitable for cross-task and cross-population comparisons [108]. Fixation count and transition metrics offered complementary insights into the organization and flexibility of gaze behavior [109]. These parameters, quantifying the number of discrete fixations or gaze shifts between AOIs, revealed distinctive patterns of visual exploration in ASD. Increased fixation counts on non-social content and fewer transitions between social targets suggest a restricted and perseverative scanning style, reflecting reduced attentional flexibility. Within joint attention and dynamic interaction tasks, lower transition frequencies between faces and objects further illustrated diminished coordination of social gaze. These findings highlight that atypical visual attention in ASD involves not only reduced engagement but also altered sequencing of gaze behavior [86].

In parallel to fixation-based findings, saccadic metrics also contributed meaningful insights into attentional dynamics in ASD. Across studies, autistic children—particularly those showing a geometric stimulus preference—exhibited reduced saccadic activity when viewing preferred non-social stimuli and increased saccadic rates when confronted with social scenes, suggesting altered attentional flexibility and atypical orienting toward socially salient information [110]. These patterns indicate that saccadic behavior may capture rapid, moment-to-moment adjustments in visual exploration and, when combined with fixation indices, enhance the sensitivity of eye-tracking–based profiles for distinguishing ASD subgroups and supporting early diagnostic classification [111].

Spatial gaze metrics provided a valuable extension of fixation-based analyses, capturing how gaze is spatially distributed across the visual field. Measures such as fixation dispersion, D2R, and gaze cohesion indices revealed greater variability and less centralized focus among autistic participants. Such spatial irregularities are consistent with fragmented or idiosyncratic viewing strategies and have been linked to symptom severity, suggesting that spatial gaze organization may serve as a sensitive marker of social-perceptual integration [112]. These findings point toward the importance of spatial metrics in quantifying not only where participants look but also how consistently and cohesively gaze is maintained [113].

Finally, derived and composite fixation indices represent an emerging methodological advancement, integrating multiple primary metrics into higher-order indicators. Approaches such as difference scores (e.g., social–object ratios), entropy-based measures, and multivariate composites like the OMI or Typicality Scores offer promising analytical tools [75,112]. However, these indices are still exploratory, are derived from small samples, and lack external validation; therefore, their diagnostic value should be viewed as preliminary rather than established [114]. These indices capture more abstract properties of visual attention, such as regularity, predictability, or similarity to normative patterns and have shown strong associations with clinical severity measures [112]. The convergence of results across diverse analytical frameworks highlights their potential for improving the precision and interpretability of eye-tracking outcomes [76].

In conclusion, fixation-based metrics—whether temporal, proportional, count-based, spatial, or derived—collectively form a multidimensional framework for characterizing visual attention atypical in ASD. Temporal and proportional measures remain the most established, while spatial and composite indices offer promising avenues for capturing the complexity of gaze organization [91]. The observed methodological heterogeneity across studies underscores the need for standardized analytic frameworks and cross-paradigm validation to fully leverage these metrics for early identification, stratification, and treatment monitoring in autism.

### 4.3. Diagnostic Accuracy, Sensitivity, and Specificity of Eye-Tracking Metrics

The body of evidence reviewed demonstrates that eye-tracking-based paradigms can distinguishing autistic children from typically developing peers, with a subset of studies reporting exploratory classification metrics, including accuracy and specificity, within the studied samples. Notably, composite models combining multiple gaze features, such as fixation duration and saccade dynamics, tend to yield higher classification performance than single-feature approaches.

However, the review also reveals significant variability in sensitivity across studies, particularly in paradigms that rely on a single behavioral marker or visual preference (e.g., the GeoPref Test). While this paradigm consistently shows high specificity (95–98%), its lower sensitivity (17–33%) limits its use as a standalone diagnostic tool. These findings suggest that visual preference tasks appear more effective for identifying specific ASD subgroups characterized by pronounced stimulus-driven attentional biases, rather than capturing the full heterogeneity of ASD presentations. Accordingly, their potential utility should be considered limited to subgroup characterization or stratification purposes, rather than as general diagnostic tools applicable across the autism spectrum. Moreover, certain paradigms, such as those focusing on spatial gaze metrics (e.g., D2R), also yielded high AUCs (>0.85), indicating that spatial coherence and gaze anchoring may be particularly sensitive markers of social attention atypicality.

Importantly, while specificity tends to be high across paradigms—indicating low false positive rates—sensitivity remains a methodological challenge. Several factors may contribute to this, including task design, sample heterogeneity, age range, and the cognitive demands of the paradigms themselves. For instance, tasks with high ecological validity or interactive content may better reflect real-world social processing but are harder to standardize. Conversely, simplified paradigms enhance standardization but may not capture more nuanced impairments. Taken together, these differences in task structure and cognitive demands contribute to the broader methodological heterogeneity observed in the eye-tracking literature.

In conclusion, eye-tracking shows clear potential as a non-invasive, objective, and scalable approach for characterizing early visual attention patterns and supporting stratification within the autism spectrum. Although all included studies aimed to explore the diagnostic relevance of eye-tracking, the evidence they provide is primarily based on group-level comparisons between autistic and typically developing children. Such designs demonstrate the presence of a discriminative signal but do not establish diagnostic validity at the individual level. Moreover, the observed discriminative performance appears highly dependent on the specific metrics and experimental paradigms employed. Future research should aim to (a) optimize task sensitivity without compromising specificity, (b) develop composite indices that combine multiple gaze features, and (c) validate these tools across diverse populations and settings.

## 5. Conclusions

Eye-tracking represents a promising research approach toward more objective and precise methods characterization of visual attention patterns associated with ASD. Across studies, eye-tracking measures have been shown to capture reproducible group-level differences between autistic and typically developing children, which may support phenotypic characterization and inform future diagnostic-validation research. In this context, eye-tracking may complement existing assessment approaches, such as parental reports, by providing objective markers of visual attention, rather than functioning as a standalone screening tool [115].

Although the studies included do not allow a meta-analysis to be performed, the findings provide valuable preliminary evidence in support the potential relevance of eye-tracking paradigms as candidate biomarkers in ASD.

Autistic individuals have consistent gaze abnormalities that highlight problems in the selection of socially relevant information. Aggregating these abnormalities across stimuli and ROIs may offer objective outcome measures for research and stratification purposes [29].

Based on this systematic review and qualitative analysis, from the complexity of all the studies included emerged the need to address several essential method issues, to elaborate more consistent and comparable data for future studies. Many studies have employed tasks involving the presentation of visual stimuli, tracking of a moving object or person, and examination of pupillary response to specific stimuli [116,117].

Although eye-tracking metrics showed promise in terms of diagnostic accuracy across studies, the high risk of bias in key QUADAS-2 domains, particularly about patient selection, application of the index test, and flow and timing, limits overall confidence in these estimates. Given the pervasive nature of these biases, reported measures of accuracy, sensitivity, and specificity are likely to be unstable and potentially inflated, and should therefore not be interpreted as robust indicators of diagnostic performance. Moreover, although pooled specificity was high, the very low pooled sensitivity (35.9%) indicates that current eye-tracking measures cannot be considered suitable for screening or standalone diagnosis, as they would miss a substantial proportion of autistic children. From a clinical perspective, this level of sensitivity represents a major limitation, as it would lead to an unacceptably high rate of false negatives, thereby undermining the practical applicability of eye-tracking for early detection or diagnostic decision-making.

## 6. Limitation

Several limitations persist across studies utilizing eye tracking as a tool for measuring or identifying potential biomarkers for autism diagnosis. These limitations include:

- Sample size: many studies have small sample sizes, which can affect the generalizability of the results. Furthermore, several studies have predominantly involved male autistic participants (M:F ≥ 5:1), which reduces the applicability of the results to women, who remain underrepresented in eye tracking research.
- Task design: different eye-tracking tasks have been used to study autism, and the task design can impact the results obtained. Task design should be carefully selected to ensure that it is relevant and appropriate for the specific population being studied.
- Eye tracking technology: eye tracking technology can also affect the results, as different technologies have different levels of accuracy and reliability.
- Interaction between gaze and other factors: gaze patterns can be influenced by many other factors, such as attention, motivation, and task demand. Importantly, the very wide age range represented in the included studies (from infancy to late adolescence) implies that the neuropsychological construct underlying visual attention to complex social scenes likely differs across developmental stages. This limits the direct comparability of results across studies, as attentional mechanisms observed at 12 months of age cannot be assumed to reflect the same cognitive processes seen in older children or adolescents.
- The high rate of high-risk trials in patient selection was probably due to the lack of a consecutive or random sample of enrolled patients. Only 2 studies ensured consecutive enrolment of patients.
- Due to the intrinsic nature of the test and the lack of a threshold, the overall risk of bias in the index test area was very high.
- Test–retest reliability to measure the consistency of results on the same sample at a different point in time. Few studies show these results and in most studies; moreover, the lack of standardized follow-up time and the inappropriate interval between the index test and the reference standard meant that only three studies achieved a low risk of bias. Only one study [75] reported test–retest reliability, and even in that case reliability was limited to specific measures. The absence of systematic reproducibility analyses across studies severely restricts the interpretability of gaze-based measures as stable traits, and highlights a major gap for any biomarker intended for clinical use.

### Future Research

Future research should systematically examine sources of heterogeneity within the autistic population. This includes identifying gaze-based subgroups, extending beyond the limited GeoPref patterns documented in a few studies, and integrating key modulators such as cognitive functioning, language level, sensory profiles, and co-occurring neuro-developmental or psychiatric conditions. Incorporating these variables into statistical models will be essential for determining whether atypical gaze reflects differences in social motivation, hyper-arousal, perceptual sensitivity, executive functioning, or other mechanisms. Only through such multimodal and developmentally informed approaches will it be possible to construct a theoretical framework that captures the diversity of autistic visual attention profiles and supports individualized diagnostic or clinical applications. Ongoing research efforts are necessary to overcome these limitations and develop standardized protocols, leading to a clinical integration of eye tracking in ASD assessment.

## Figures and Tables

**Table 4:** Eye Tracker Experimental Parameter.

Author	Neuropsychological Construct	Category	Parameters	Tipe of Stimuly	Task Type	Result	Diagnostic Accuracy
Anderson, C.J. et al. [79]	Preferential looking	Temporal Metrics	Total fixation time; fixation duration; mean fixation duration	Static Social & Not-social Passive	Children passively looked at still images for 15 s each while seated; no explicit behavioral response required.	Children with ASD showed reduced fixation time and shorter fixation durations on landscapes compared to TD. These visual scanning measures correlated negatively with the ADOS-G Behavior subscale (r ≈ −0.76, *p* < 0.05), indicating less exploration was linked to greater behavioral impairment. No significant group differences for fixation on faces or internal facial regions	Visual scanning measures alone (time tracked, fixation duration on landscapes) significantly differentiated ASD from both control groups. In discriminant analysis, these scanning variables contributed strongly to correct group classification (part of 78% overall accuracy)
Chevallier, C. et al. [67]	preferential looking paradigm; activity monitoring paradigm	Temporal Metrics; Proportion Metrics; Derived Metrics;	Total fixation time; fixation proportion; fixation difference	Dynamic & Static Social & Not-social Passive—Active	(I) “Static Visual Exploration” task displayed static images of objects and people. (II) In the “Dynamic Visual Exploration” task simultaneously played four dynamic video clips of individual faces and objects. (III) An “Interactive Visual Exploration” task presented highly ecological video clips of children playing together.	The Interactive task was the only one that differentiated between the ASD and the TD group. The ASD group spent less time looking at social stimuli, and marginally more time looking at object stimuli.	AUC = 0.721 (*p* = 0.002) for the Interactive task; no significant AUC for Static or Dynamic tasks.
Falck Ytter, T. [68]	preferential looking paradigm	Temporal Metrics; Proportion Metrics; Derived Metrics	Total fixation time; fixation proportion; fixation correlation	Dynamic Social Passive	The children watched short videos on a screen consisting of 24 clips, with a model showing 6 expressions × in an upright/upside-down position.	Both groups looked less at faces during inverted presentations (*p* = 0.005). ASD children showed less change in fixation pattern (upright vs. inverted). Positive correlations between eye/mouth fixations across conditions only in ASD (r = 0.62–0.71, *p* < 0.05).	No AUC or cut-off reported. However, fixation metrics significantly differentiated ASD from TD (t(28) = −2.19, *p* = 0.037).
Falck Ytter, T. et al. [69]	social information processing	Temporal Metrics; proportion Metrics; count metrics; Spatial metrics; Derived metrics	Total fixation time; fixation proportion; number of fixation; fixation rate; fixation distance (D2R);	Dynamic Social & Non-Social Passive	The stimuli were six short 20 s videos of semi-naturalistic social interactions between two young children (one slightly older than the other), shown in different orders to each participant. The reference point (r) was the nose of one actors.	In all tasks, a significantly different fixation duration was identified between the groups in the first 4–6 s. During this period, children with TD tended to focus on the actor’s face, whereas this tendency was weaker and delayed in children with ASD. In all tasks, a significant difference in D2R values was observed between the groups in the first 4–6 s. In addition, other time intervals were identified in which there were group differences in D2R, but during these periods the groups did not differ significantly in fixation duration.	AUC = 0.916 (AOI), AUC = 0.855 (corrected AOI), AUC = 0.815 (D2R)
Frazier, T.W. et al. [76]	Social information processing paradigm	Temporal metrics; Proportion metrics; count metrics;	Number of fixation; fixation proportion; fixation duration, total fixation time; mean fixation duration	Dynamic & Static Social & Not-social Passive	Passive viewing of 44 stimuli including faces, joint attention, social bids, abstract shapes, and naturalistic interactions	The Autism Risk Index (ARI) showed excellent discriminative validity for ASD diagnosis. Fixation metrics derived from temporal ROIs significantly contributed to this index. Although no direct correlation with ADOS-2 severity scores was observed.	AUC: 0.92 (train), 0.86 (test); AUC < 4y = 0.925, ≥4y = 0.931; ΔR^2^ = 0.22 vs. ADOS-2; ASI–ADOS-2 correlation: r = 0.58–0.67
Jones, W. et al. [80]	preferential looking paradigm	Temporal metrics; Proportion metrics; Saccade metrics	fixation proportion; total fixation time, Saccade velocity	Dynamic Social & Non-Social Active	All videos showed actresses playing the role of a caregiver, looking directly into the camera, and entreating the viewing child by engaging in childhood games (with both video and audio).	Autistic subjects stared significantly less on the eye region and more on the mouth region than the TD and DD.	Fixation to eyes was the strongest predictor of group membership (Cohen’s d = 1.56 vs. TD; d = 1.40 vs. DD). Eye fixation was significantly correlated with social disability (r = −0.669, *p* < 0.01)
Kou, J. et al. [70]	preferential looking paradigm	Temporal Metrics; Proportion metrics; Derived Metrics	Total fixation time; fixation duration; fixation proportion; fixation difference	Dynamic & Static Social & Non-Social Passive	(1) The first task displays dynamic dancing Chinese humans versus dynamic geometry patterns. (2) The second task displays point-light animate (walking human or cat) and inanimate (randomly moving point-light) videos. (3) The last task compared attention to static pictures showing a toy alone or with a child playing with it.	The ASD group spent significantly more total time and more fixation counts on geometric images than TD. %Total fixation duration and count are significantly sensitive in task (1) and task (3), or combining them.	ROC AUC for Task 1 (DSI): 0.801 (fixation duration), 0.815 (fixation count); Task 2 (cat animation): 0.742; Task 3 (toy with child): 0.714. Best association with symptom severity: Task 1, ADOS-2 SA (r = −0.45 to −0.52).
Muratori, F. et al. [77]	joint attention paradigm	Temporal metrics; Proportion metrics; Derived metrics; Count metrics	Number of transitions; fixation difference (normalized transition score); fixation duration; fixation proportion;	Dynamic Social & Non-Social Passive	IJA-1 with a predictable event: an actress was positioned between two little cars placed on the table in front of her and one of the two cars (‘target object’) moved, while the actor maintained a direct gaze to the child with a neutral expression; IJA-2 with an unpredictable event: the same actress was initially alone in the scene, and then a toy truck (“target object”) appeared unexpectedly and crossed the screen while the actress maintained a direct gaze with a neutral expression.	In IJA-1, ASD had significantly higher transitions from target object to face, and significantly higher normalized transition scores compared to TD. In the IJA-2 task, ASD had significantly higher transitions from target object to face and from face to target object than TD. Moreover, ASD had a significantly higher fixation duration to face. Six months later in the IJA2 task, ASD differences in transitions from face to target object were still higher.	Eye-tracking metrics predicted longitudinal clinical changes. Specifically, ADOS items at baseline (e.g., pointing, gesturing, showing) significantly predicted visual attention changes at follow-up. Regression models showed moderate predictive power (e.g., ADOS_A7 → transitions from face to non-target object: β = −0.63, adj-R^2^ = 0.34, *p* = 0.027).
Pierce, K. et al. [71]	preferential looking paradigm	Temporal metrics; Proportion metrics; Derived metrics; saccades metrics	total fixation time; fixation proportion; fixation difference; saccade count	Dynamic Social & Non-Social Passive	A video simultaneously reproduced geometric (DGI) and social images (DSI). The DGI consisted of animated screen saver programs. The DSI consisted of a series of short sequences of children doing yoga. The final movie contained a total of 28 scenes with single-scene duration varying from 2 to 4 s for a total presentation time of 60 s at 24 frames per second.	Toddlers with ASD spent significantly more time fixating DGI compared to TD toddlers and toddlers with DD. ASD who preferred geometric images showed significantly fewer saccades per second while viewing those and higher saccade rates while viewing non-preferred social images.	ROC curve analysis: AUC = 0.686 (*p* < 0.001). Using a cutoff of 68.6% fixation time on geometric patterns yielded a Positive Predictive Value (PPV) of 100% for ASD diagnosis.
Polzer, L. et al. [72]	preferential looking paradigm; changing light condition paradigm	Temporal metrics; Proportional metrics; derived metrics	Fixation proportion; fixation duration; fixation difference	Dynamic & Static Social & Not-Social Passive	(a) simultaneous presentation of one geometric motion video and social motion video, for 10 different trials, each slide lasting 6 s; (b) Black and white slides were presented alternately on the screen for a total of 12 trials, each slide lasted 5 s.	The attenuated preference for social movement in the ASD group compared with the TD group statistically predicted the diagnosis. ASDs showed attenuated SEPR in response to social stimuli and attenuated LAPR to dark light conditions. Only SEPR was associated with social motion preference.	Predictive model using SOC-M preference (pseudo R^2^ = 0.486)
Putra, P et al. [73]	Inhibiting action and spatial and gaze-adjustment	Temporal metrics; spatial metrics; derived metrics	Total Fixation time; fixation distance; gaze velocity; gaze acceleration; gaze entropy; gaze adjustment coefficient	Static Not-Social Active	The game represented the Go and NoGo stimuli as “Chicken” and “Cat” characters, respectively. A stimulus appeared randomly in one of nine locations for a fixed duration of time. The children should respond to the chicken character (Go stimulus) by pressing a space bar but they must inhibit their action towards the cat character (NoGo stimulus). The system categorized a subject’s response as one of four types: Go-positive if the subject responded to the Go character; Go-negative if they missed it; NoGo-positive if they inhibited their action in response to the NoGo character; NoGo-negative if they reacted to it.	A statistically significant difference in spatial and auto-regressive temporal gaze-related features.	AUC > 0.85 Accuracy = 88.6% MCC supports good specificity, though no numeric value reported.
Shic, F. et al. [75]	(I, II, III) Activity Monitoring, (IV) preferential looking paradigm	Proportion metrics; Derived metrics	fixation proportion; fixation composite index; fixation difference	Dynamic & Static Social & Not- Social Passive	Four of these tasks focused on social-attentional constructs and included: (I) Activity Monitoring depicting videos of two adults playing with toys; (II) the Social Interactive task, videos of two children engaged in parallel and joint play; (III) Static Social Scenes (StaticScenes), images depicting varied naturalistic scenes involving people; and (IV) Biological Motion Preference, point-light display videos of biological motion versus non-biological control stimuli shown side-by-side. During static image trials, a wordless soundtrack was played. During video trials, the actresses spoke in child-friendly language and directed their eyes to each other (mutual gaze) or the joint activity (activity gaze).	ASD had lower OMI scores, and looked less at faces in ActivityMonitoring, SocialInteractive, and StaticScenes tasks, confirmed also on test–retest reliability. The ASD group had later Pupillary light reflex latencies but did not persist on short-term stability.	Group discrimination supported (OMI: Cohen’s d = 0.788; η^2^ = 0.117); not diagnostic but useful for subgroup stratification
Wang, Q. et al. [78]	Activity Monitoring; joint attention paradigm	Derived metrics; spatial metrics	fixation index; fixation typically index; cohesion value; proportion of high cohesion frame;	Dynamic Social & Non-Social Passive	The stimuli were shown a 3 min video with four conditions in which an actress looks directly at the camera to elicit eye contact or to elicit joint attention turning by looking at a toy with different modalities (Dyadic Bid, Sandwich, Joint Attention, and Animated Toys).	The ASD group had significantly lower typicality scores compared to the other group in Sandwich and Dyadic Bid.	Significant group differences in gaze typicality scores across conditions; lower scores in ASD associated with greater symptom severity (ADOS-SA). Pseudo-R^2^ = 0.486.
Wen, T. H et al. [73]	preferential looking paradigm	Proportion metrics; derived metrics; saccade metrics	fixation proportion; fixation difference; saccade count and frequency	Dynamic Social & Non-Social Passive	GeoPref eye-tracking test, composed of a series of short sequences of 2 rectangular areas of interest containing dynamic social (DSI) and geometric (DGI) images.	Children with ASD showed the highest percentage of fixation duration at DGI compared to other sample groups: the percentage of DGI fixation was significantly correlated with all clinical measures and all associated subscales. ASD who preferred geometric images showed significantly fewer saccades per second while viewing those and higher saccade rates while viewing non-preferred social images; ASD who preferred social images had nearly typical saccadic patterns. Combining these two parameters, the GeoPref Test had 98% specificity, 33% sensitivity, 81% PPV, and 65% NPV.	GeoPref Test (fixation ≥ 69%): Spec 98%, Sens 17%, PPV 81%, NPV 65%; GeoPref + saccades/s (fix ≥ 61.3%, saccades/s ≥ 2.29): Spec 95.2%, Sens 33.3%, Acc 71%, PPV 81.4%, NPV 71.2%

## Data Availability

No new data were created or analyzed in this study.

## Supplementary Materials

The following supporting information can be downloaded at: https://www.mdpi.com/article/10.3390/medsci14010028/s1, Table S1: PRISMA DTA-checklist; Table S2: Risk of bias (quality) assessment; Supplement S1: Literature search; Supplement S2: QUADAS-2 specific protocol template.

## Abbreviations

The following abbreviations are used in this manuscript:

ASD	Autism Spectrum Disorder
TD	Typically developing
AoI	Ares of Interest
ICD-10	International Classification of Diseases
ADOS	Autism Diagnostic Observation Schedule
ADI-R	Autism Diagnostic Interview-Revised
MSEL	Mullen Scales of Early Learning
VABS	Vineland Adaptive Behavior Scales
DISCO	Diagnostic Interview for Social and Communication Disorders
M-CHAT	Modified Checklist for Autism in Toddlers
ABC	Aberrant Behavior Checklist
RoI	Region of Interest
DGI	Dynamic Geometric Images
SOC-M	Social Motion
GEO-M	Geometric Motion
OMI	Oculomotor Index of Gaze to Human Faces
D2R	Distance-to-Reference
AUC	Area Under the Curve
ROC	Receiver Operating Characteristic
QUADAS	Quality Assessment of Diagnostic Accuracy Studies
PRISMA	Preferred Reporting Items for Systematic Reviews and Meta-Analyses
DTA	Diagnostic Test Accuracy
