# Supplementary material for: The Diagnostic Potential of Eye Tracking to Detect Autism Spectrum Disorder in Children: A Systematic Review
Source: Med Sci (Basel). 2026 Jan 6;14(1):28. doi: 10.3390/medsci14010028 (PMC12821402; doi:10.3390/medsci14010028)
Supplement: Supplementary file 1 [file medsci-14-00028-s001.zip › medsci-4008559-supplementary.pdf]

SUPPLEMENTARY MATERIAL

Table S1: PRISMA DTA-checklist.…..…..…..…..…..…..…...…..…..…..……....…..…..……..…..…..……………………..page 2-4

Table S2: Risk of bias (quality) assessment.…...…..…...…..…..…....…..…..…..…..……..…..…..…..…..…...…..…....page 5

Supplement 1: Literature search..…..…...…....…..…...…....…..…...……….…...…...…..…...…...…..…...…...…..…...……page 6

Supplement 2: QUADAS-2 specific protocol template …………………………………………………………………...page 7-14

This supplementary material has been provided by the authors to give readers additional information about their work and provides methodological documentation for the meta-analysis of diagnostic accuracy presented in the manuscript.

Table S1: PRISMA-DTA Checklist Item

Section/topic	#	PRISMA-DTA Checklist Item	Reported on page #
**TITLE / ABSTRACT**			
Title	1	Identify the report as a systematic review (+/- meta-analysis) of diagnostic test accuracy (DTA) studies.	Title
Abstract	2	Abstract: See PRISMA-DTA for abstracts.	Abstract
**INTRODUCTION**			
Rationale	3	Describe the rationale for the review in the context of what is already known.	Introduction
Clinical role of index test	D1	State the scientific and clinical background, including the intended use and clinical role of the index test, and if applicable, the rationale for minimally acceptable test accuracy (or minimum difference in accuracy for comparative design).	Introduction
Objectives	4	Provide an explicit statement of question(s) being addressed in terms of participants, index test(s), and target condition(s).	Introduction
**METHODS**			
Protocol and registration	5	Indicate if a review protocol exists, if and where it can be accessed (e.g., Web address), and, if available, provide registration information including registration number.	Methods
Eligibility criteria	6	Specify study characteristics (participants, setting, index test(s), reference standard(s), target condition(s), and study design) and report characteristics (e.g., years considered, language, publication status) used as criteria for eligibility, giving rationale.	Methods (Selection Criteria)
Information sources	7	Describe all information sources (e.g., databases with dates of coverage, contact with study authors to identify additional studies) in the search and date last searched.	Methods (Information sources and search strategy)
Search	8	Present full search strategies for all electronic databases and other sources searched, including any limits used, such that they could be repeated.	Methods (Supplement 1_ Literature search)

Study selection	9	State the process for selecting studies (i.e., screening, eligibility, included in systematic review, and, if applicable, included in the meta-analysis).	Methods (Selection procedures)
Data collection process	10	Describe method of data extraction from reports (e.g., piloted forms, independently, in duplicate) and any processes for obtaining and confirming data from investigators.	Methods (Selection procedures)
Definitions for data extraction	11	Provide definitions used in data extraction and classifications of target condition(s), index test(s), reference standard(s) and other characteristics (e.g. study design, clinical setting).	Methods (Data extraction)
Risk of bias and applicability	12	Describe methods used for assessing risk of bias in individual studies and concerns regarding the applicability to the review question.	Methods (Quality assessment)
Diagnostic accuracy measures	13	State the principal diagnostic accuracy measure(s) reported (e.g. sensitivity, specificity) and state the unit of assessment (e.g. per-patient, per-lesion).	Methods
Synthesis of results	14	Describe methods of handling data, combining results of studies and describing variability between studies. This could include, but is not limited to: a) handling of multiple definitions of target condition. b) handling of multiple thresholds of test positivity, c) handling multiple index test readers, d) handling of indeterminate test results, e) grouping and comparing tests, f) handling of different reference standards	

Section/topic	#	PRISMA-DTA Checklist Item	Reported on page #
Meta-analysis	D2	Report the statistical methods used for meta-analyses, if performed.	n.a
Additional analyses	16	Describe methods of additional analyses (e.g., sensitivity or subgroup analyses, meta-regression), if done, indicating which were pre-specified.	Methods (Quality assessment)
**RESULTS**			
Study selection	17	Provide numbers of studies screened, assessed for eligibility, included in the review (and included in meta-analysis, if applicable) with reasons for exclusions at each stage, ideally with a flow diagram.	Results, Figure 1

Study characteristics	18	For each included study provide citations and present key characteristics including: a) participant characteristics (presentation, prior testing), b) clinical setting, c) study design, d) target condition definition, e) index test, f) reference standard, g) sample size, h) funding sources	Results, Table 1, Table 2, Table 3, Table 4
Risk of bias and applicability	19	Present evaluation of risk of bias and concerns regarding applicability for each study.	Results (studies quality, Table s2)
Results of individual studies	20	For each analysis in each study (e.g. unique combination of index test, reference standard, and positivity threshold) report 2x2 data (TP, FP, FN, TN) with estimates of diagnostic accuracy and confidence intervals, ideally with a forest or receiver operator characteristic (ROC) plot.	Results
Synthesis of results	21	Describe test accuracy, including variability; if meta-analysis was done, include results and confidence intervals.	Results
Additional analysis	23	Give results of additional analyses, if done (e.g., sensitivity or subgroup analyses, meta-regression; analysis of index test: failure rates, proportion of inconclusive results, adverse events).	Results (studies quality)
**DISCUSSION**			
Summary of evidence	24	Summarize the main findings including the strength of evidence.	Discussion
Limitations	25	Discuss limitations from included studies (e.g. risk of bias and concerns regarding applicability) and from the review process (e.g. incomplete retrieval of identified research).	Limitations
Conclusions	26	Provide a general interpretation of the results in the context of other evidence. Discuss implications for future research and clinical practice (e.g. the intended use and clinical role of the index test).	Conclusions
**FUNDING**			
Funding	27	For the systematic review, describe the sources of funding and other support and the role of the funders.	Funding

Adapted From: McInnes MDF, Moher D, Thombs BD, McGrath TA, Bossuyt PM, The PRISMA-DTA Group (2018). Preferred Reporting Items for a Systematic Review and Meta-analysis of Diagnostic Test Accuracy Studies: The PRISMA-DTA Statement. JAMA. 2018 Jan 23;319(4):388-396. doi: 10.1001/jama.2017.19163.

Table S2: QUADAS-2 risk of bias and applicability summary. QUADAS-2 risk of bias and applicability summary. This is a summary of the risk of bias and applicability concerns identified in the 15 included studies, as assessed using the QUADAS-2 tool. The domains assessed were patient selection, the index test, the reference standard, and flow and timing.

Study	RISK OF BIAS				APPLICABILITY CONCERNS		
	PATIENT SELECTION	INDEX TEST	REFERENCE STANDARD	FLOW AND TIMING	PATIENT SELECTION	INDEX TEST	REFERENCE STANDARD
Anderson, 2006	☹	?	☺	☹	☹	☺	☺
Chevalier, 2015	☹	☹	☺	?	☺	☺	☺
Falk Ytter, 2008	☹	?	☺	☹	☹	☺	☺
Falk Ytter, 2013	☹	☹	☹	?	☺	☺	☺
Frazier, 2018	☺	☹	☺	☺	☺	☺	☺
Jones, 2008	☹	☹	☺	?	☺	☺	☺
Kou, 2019	☹	☹	☹	?	☺	☺	☺
Muratori, 2019	☹	☹	☺	?	☺	☺	☺
Pierce, 2011	☹	☺	☺	☺	?	☺	☺
Polzer, 2022	☹	☹	☺	?	☺	☺	☺
Putra, 2021	☹	☹	☺	☹	☹	☺	☺
Shic, 2022	☹	☹	☺	☹	☹	☺	☺
Wang, 2018	☹	☹	☺	☹	☺	☺	☺
Wen, 2022	☺	☹	☺	☺	☺	☺	☺

☺ Low Risk ☹ High Risk ? Unclear Risk

Supplement 1: Literature search

Two seasoned information specialists (MDC and CDD) crafted the search strategy in accordance with the PRISMA guideline for reporting. Keywords were carefully curated based on expert opinions. The following electronic databases and international trial registries were searched PubMed, Web of Science, and Science Direct. Search syntax for each database (in alphabetical order)

A. **PUBMED**

The search strategy was defined by identifying four mash terms related to: ("autism s"[All Fields] OR "autisms"[All Fields] OR "autistic disorder"[MeSH Terms] OR ("autistic"[All Fields] AND "disorder"[All Fields]) OR "autistic disorder"[All Fields] OR "autism"[All Fields]) AND ("diagnosable"[All Fields] OR "diagnosi"[All Fields] OR "diagnosis"[MeSH Terms] OR "diagnosis"[All Fields] OR "diagnose"[All Fields] OR "diagnosed"[All Fields] OR "diagnoses"[All Fields] OR "diagnosing"[All Fields] OR "diagnosis"[MeSH Subheading]) AND ("eye tracking technology"[MeSH Terms] OR ("eye tracking"[All Fields] AND "technology"[All Fields]) OR "eye tracking technology"[All Fields] OR ("eye"[All Fields] AND "tracking"[All Fields]) OR "eye tracking"[All Fields])

B. SCIENCE DIRECT

The search strategy was defined by identifying four mesh terms related to: ("autism" OR "autism spectrum disorders" OR "ASD") AND “diagnosis” AND "eye tracking".

C. WEB OF SCIENCE

The search strategy was defined by identifying four mash terms related to: *((ALL=(autism)) AND ALL=(diagnosis)) AND ALL=(eye tracking)*

Supplement 2: QUADAS-2 specific protocol template

Phase 1: State the review question

**Patients (setting, intended use of index test, presentation, prior testing): ***	Children and adolescents (0–18 years) suspected or diagnosed with Autism Spectrum Disorder (ASD), recruited in research, clinical, or educational settings. The intended use of the index test (eye-tracking) is to support early detection or phenotypic characterisation of ASD by assessing visual attention patterns. Participants may or may not have undergone prior testing with gold-standard tools such as ADOS-2 or ADI-R.
**Index test(s)**	Eye-tracking paradigms designed to measure gaze behaviour, visual attention, and oculomotor responses in children with suspected or diagnosed Autism Spectrum Disorder. Tests include tasks such as preferential looking, joint attention, free-viewing of social/non-social stimuli, and dynamic scene analysis. The index tests vary in terms of hardware, sampling rate, stimuli type, and outcome metrics (e.g., fixation duration, saccadic latency, gaze alternation, social/non-social fixation ratio).
**Reference standard and target condition**	Standardised clinical diagnostic instruments for Autism Spectrum Disorder, such as the Autism Diagnostic Observation Schedule, Second Edition (ADOS-2), and/or the Autism Diagnostic Interview – Revised (ADI-R), administered by trained professionals. In some studies, clinical diagnosis was based on multidisciplinary assessment following DSM-5 criteria, with or without standardised tools.<br>Autism Spectrum Disorder (ASD), as defined by the Diagnostic and Statistical Manual of Mental Disorders (DSM-IV or DSM-5), characterised by persistent deficits in social communication and interaction, along with restricted, repetitive patterns of behaviour, interests, or activities.

* Setting, intended use of the test in the clinic, clinical presentation, any previous tests

Phase 2: Draw a flow diagram for the primary study

- Participant Identification: Children (toddlers to adolescents) are recruited from clinical, research, or educational settings, with suspected or confirmed ASD or as typically developing (TD) controls.
- Eligibility and Consent: Participants are screened for inclusion/exclusion criteria; informed consent is obtained from parents or guardians.
- Reference Standard Assessment: ASD diagnosis is confirmed using a gold-standard diagnostic instrument (e.g., ADOS-2, ADI-R), or by clinical evaluation based on DSM-5 criteria.
- Index Test (Eye-Tracking): Participants complete one or more eye-tracking tasks designed to assess visual attention (e.g., preferential looking, joint attention, free-viewing). Data are collected using infrared-based or screen-based eye-tracking devices.
- Outcome Assessment: Eye-tracking metrics (e.g., fixation duration, gaze alternation, saccadic patterns) are extracted and analysed. Some studies may report group comparisons (ASD vs TD), while others provide accuracy measures (e.g., AUC, sensitivity, specificity).
- Analysis and Classification: Studies explore associations between eye-tracking features and diagnostic categories or compute classification performance (in studies attempting predictive modelling).

Phase 3: Risk of bias and considerations on applicability

QUADAS-2 is structured into four domains: patient selection, study tests, reference standards, flow and timing. The risk of bias is assessed for each domain, and applicability is also assessed for the first three domains. Guiding questions are included to help guide the assessment of risk of bias.

**DOMAIN 1. PATIENT SELECTION**			
**A. Risk of Bias**			
Describe methods of patient selection:			
Was a consecutive or random sample of patients enrolled?	Yes	No	Unclear
Was a case-control design avoided?	Yes	No	Unclear
Did the study avoid inappropriate exclusions?	Yes	No	Unclear

**Could the selection of patients have introduced bias?**	Low risk	High risk	Unclear risk
**B. Concerns regarding applicability**			
Describe included patients (prior testing, presentation, intended use of index test and setting):			
**Is there concern that the included patients do not match the review question?**	Low risk	High risk	Unclear risk

DOMAIN 2: INDEX TEST(S) If more than one index test was used, please complete for each test.			
**A. Risk of Bias**			
Describe the index test and how it was conducted and interpreted:			
Were the index test results interpreted without knowledge of the results of the reference standard?	Yes	No	Unclear
If a threshold was used, was it pre-specified?	Yes	No	Unclear
**Could the conduct or interpretation of the index test have introduced bias?**	Low risk	High risk	Unclear risk
**B. Concerns regarding applicability**			

**Is there concern that the index test, its conduct, or interpretation differ from the review question?**	Low risk	High risk	Unclear risk

**DOMAIN 3: REFERENCE STANDARD**			
**A. Risk of Bias**			
Describe the reference standard and how it was conducted and interpreted:			
Is the reference standard likely to correctly classify the target condition?	Yes	No	Unclear
Were the reference standard results interpreted without knowledge of the results of the index test?	Yes	No	Unclear

**Could the reference standard, its conduct, or its interpretation have introduced bias?**	Low risk	High risk	Unclear risk
**B. Concerns regarding applicability**			
**Is there concern that the target condition as defined by the reference standard does not match the review question?**	Low risk	High risk	Unclear risk

**DOMAIN 4: FLOW AND TIMING**
**A. Risk of Bias**
Describe any patients who did not receive the index test(s) and/or reference standard or who were excluded from the 2x2 table (refer to flow diagram):<br><br>Describe the time interval and any interventions between index test(s) and reference standard:

Was there an appropriate interval between index test(s) and reference standard?	Yes	No	Unclear
Did patients receive the same reference standard?	Yes	No	Unclear
Were all patients included in the analysis?	Yes	No	Unclear
**Could the patient flow have introduced bias?**	Low risk	High risk	Unclear risk
